# The epidemiology of listeriosis in pregnant women and children in New Zealand from 1997 to 2016: an observational study

**DOI:** 10.1186/s12889-020-8221-z

**Published:** 2020-01-28

**Authors:** Emma Jeffs, Jonathan Williman, Cheryl Brunton, Joanna Gullam, Tony Walls

**Affiliations:** 1 Department of Paediatrics, University of Otago, PO Box 4345, Christchurch Mail Centre, Christchurch, 8140 New Zealand; 2 Department of Population Health, University of Otago, PO Box 4345, Christchurch Mail Centre, Christchurch, 8140 New Zealand; 3 Department of Obstetrics and Gynaecology, University of Otago, PO Box 4345, Christchurch Mail Centre, Christchurch, 8140 New Zealand

**Keywords:** *Listeria monocytogenes*, Listeriosis, Pregnant, Pregnancy, Neonate, Infant, Child

## Abstract

**Background:**

*Listeria monocytogenes* causes the foodborne infection listeriosis. Pregnant women, infants and immunocompromised children are at increased risk for infection. The aim of this study was to describe the trends in the epidemiology of disease notifications and hospital admissions due to listeriosis in pregnant women aged 15 to 45 years and children aged less than 15 years in New Zealand (NZ) from 1997 to 2016.

**Methods:**

In this population-based descriptive study, listeriosis notification and hospitalization rates from 1997 to 2016 were analyzed. Notification data were extracted from the Institute of Environmental Science and Research (ESR) Notifiable Diseases Database (EpiSurv) and hospitalization data were extracted from the National Minimum Dataset (NMDS). Pregnant women aged 15 to 45 years and children less than 15 years of age were included. Subgroup analysis was conducted for age and ethnicity. Outcomes of infection were described.

**Results:**

In the 20-year period considered, there were 147 pregnancy-associated cases of listeriosis either notified to ESR (*n* = 106) and/or coded in the NMDS (*n* = 99), giving a crude incidence rate of 12.3 (95% CI 10.4, 14.4) per 100,000 births. In addition, there were 22 cases in children aged 28 days to < 15 years (incidence =0.12, 95% CI 0.08 to 0.19 per 100,000). There were no trends observed over time in the incidence of pregnancy-associated listeriosis. Incidence rates of pregnancy-associated and childhood listeriosis were highest in people of Pacific and Asian ethnicity.

**Conclusions:**

NZ has a low incidence of listeriosis in pregnant women and children, however, the consequences of infection are frequently severe. Those of Pacific and Asian ethnicity have the highest rates of disease and future messaging around food safety should target these groups. This study provides important insights into the epidemiology of listeriosis in pregnant women and children in NZ.

## Background

*Listeria monocytogenes,* a facultative rod-shaped Gram positive bacteria, causes listeriosis*,* a serious and potentially life-threatening infection that is principally transmitted by the consumption of contaminated food [1]. The genus *Listeria* is composed of 17 species, of which *Listeria monocytogenes* is an opportunistic foodborne pathogen of humans and animals [2]. Both sporadic episodes and large outbreaks of disease have been attributed to this pathogen [3–6]. In developed countries, *L. monocytogenes* has the highest case-fatality rate of any foodborne pathogen [7].

*L. monocytogenes* has a predilection to infect pregnant women, neonates, those who are immunocompromised and the elderly. Pregnant women, in particular, are at approximately 18 times greater risk for infection than the general population owing to the natural immunosuppression of pregnancy [8]. While maternal illness is usually mild, neonatal illness is frequently severe and potentially fatal [1, 9]. Neonatal listeriosis may occur by vertical transmission of *L. monocytogenes* from mother to foetus, either by inhalation of infected amniotic fluid, trans-placentally from the maternal circulation, or by ascending colonization during birth [10].

Clinical outcomes are influenced by the gestation at which infection occurs. Listeriosis most commonly presents in the third trimester of pregnancy (from 28 weeks) and is rarely fatal in the mother, particularly in the absence of coexisting medical conditions [11, 12]. Later infection, particularly that within the third trimester, is typically associated with more favourable foetal outcomes than earlier infection [13]. If transmission to the foetus does occur, infection can lead to miscarriage, pre-term delivery or stillbirth. In a recent study of 107 cases of pregnancy-related listeriosis in France, the transmission of infection from the mother to the foetus occurred in 96% of cases, and major foetal or neonatal complications were observed in 83% of infants of infected mothers [7].

Neonatal listeriosis typically manifests within the first 24 to 72 h of life and can present as bacteremia, respiratory distress, meningitis and, less frequently, pneumonia [14]. Late-onset listeriosis, that presenting in infants aged one to 4 weeks, is most often associated with meningitis [15]. About half the infants who are infected with *L. monocytogenes* have no apparent immunocompromising condition [16]. Neonatal listeriosis is associated with an overall case fatality rate of 50% [17], with severe neurological and developmental sequalae observed in 40% of surviving neonates [18]. Due to the severity of the illness, in New Zealand (NZ) all neonates with suspected infection are treated with amoxicillin which targets *L. monocytogenes,* as well as other infections [19]. In children who are immunocompetent, listeriosis is most likely to present as an influenza-like illness or, if the infection is significant, gastroenteritis. However, in children who are immunocompromised, infection can present as several clinical syndromes, of which meningitis and bacteraemia are the most common [1].

In NZ, the Ministry of Health (MoH), the principal advisor to the NZ government on health and disability, supports public health messages aimed at reducing the risk for infection of listeriosis in pregnancy. The Ministry for Primary Industries, the ministry charged with overseeing NZ’s primary industries (e.g. farming) and food safety, publishes food safety in pregnancy guidelines which are available on-line and as a printed booklet [20]. Listeriosis has been a notifiable disease in NZ since 1983, requiring notification of laboratory-diagnosed cases to the local public health service, and food safety guidelines have been available since at least 1997.

*L. monocytogenes* infection is rare in NZ. In 2018, there were 0.6 cases per 100,000 in the population notified [21]. It is estimated that almost 90% of cases are due to foodborne transmission [22]. This is comparable to rates reported internationally. Listeriosis incidence worldwide ranges between 0.1 and 11.3 cases per 100,000 population per year [23]. While the crude incidence rates of listeriosis for the total NZ population are known, listeriosis rates in pregnant women and children have not been systematically studied. The aim of this study was to describe the trends in the epidemiology of disease notifications and hospital admissions due to listeriosis in pregnant women aged 15 to 45 years and children aged less than 15 years in NZ from 1997 to 2016.

## Methods

### Data sources

Case data were sourced from two databases of routinely collected information for this population-based descriptive study: the national notifiable disease surveillance database (EpiSurv) operated by The Centre for Environmental Science and Research Ltd. (ESR) and The National Minimum Dataset (NMDS) held by the NZ MoH.

EpiSurv contains data on notifiable diseases and should, theoretically, contain all cases of notifiable disease resulting in hospital admission, as well as cases managed in the community without hospitalization. ESR commenced standardized on-line reporting in 1997 and therefore 1997 was elected as the start point for data analysis. Laboratory definitive evidence for a confirmed case of listeriosis requires identification of *L. monocytogenes* from a normally sterile site, including the foetal gastrointestinal tract, by either isolation (culture) of *L. monocytogenes* or detection of *L. monocytogenes* nucleic acid [24]. Only invasive disease is notifiable. Where illness has occurred in a pregnant woman, foetus or infant aged ≤28 days, the mother is notified as the case and the disease is recorded as pregnancy-associated.

The NMDS is a national dataset of public and private hospital discharge information that describes clinical data for inpatients and day patients, and is coded according to international standards, including the International Classification of Diseases – Clinical Modification 9 (ICD-9-CM). The original NMDS was implemented in 1993 and back-loaded with public hospital discharge information from 1988. The NMDS (hospital events) dataset used in this study contains information on the number of episodes of disease resulting in hospital admission.

Data were requested from both databases for all listeriosis cases (ICD-9-CM code 027.0) from 1 January 1997 to 31 December 2016 for all women of childbearing age, defined as 15 to 45 years (inclusive), and all children < 15 years of age. Children < 15 years were included as this is the New Zealand cut off for pediatric hospital admissions. Data were included where listeriosis was either the principal diagnosis or another relevant diagnosis. Data were not stratified by, or excluded due to, listeria serotype, owing to variations and inconsistencies in data reporting.

Data from the NMDS were identifiable by patient National Health Index (NHI) number, a unique identifier that is assigned to every person who uses health and disability support services in NZ. All children were included, irrespective of their predicted immune status (i.e. children with known immunocompromising conditions, such as leukemia, were included and were identified using the ICD-9-CM codes: 204.01 - Acute lymphoid leukemia, in remission, and 204.00 - Acute lymphoid leukemia, without mention of having achieved remission). Women were considered pregnant if they had any ICD-9 diagnosis codes starting with V22, V23, V27, V28, or 630 to 679 (codes identifying pregnancy) in the NMDS, or if the notification in ESR was recorded as being pregnancy-associated or pregnancy was indicated in any of the text fields.

Data extracted from EpiSurv included: disease name, report date, sex, age, date of birth, ethnicity, District Health Board (of admission), mortality status (including if from confirmed listeriosis or not), meningitis status, septicaemia status, swab/culture site, recent overseas travel (confirmed or not) and current immunosuppressant drug use. For pregnancy-associated cases, data also included delivery date and pregnancy outcomes (gestation, preterm delivery, and death of foetus or infant).

The data extracted from the NMDS included: event date, admission type (acute or planned), date of birth, gender, ethnic group (prioritized), domicile, District Health Board domicile, facility (of admission) and diagnosis (according to ICD-9 code and including up to 30 diagnosis codes). For children, data also included admission weight, birth weight and gestation at birth.

For calculation of incidence rates, denominator data was sourced from the NZ MoH and Statistics NZ. The National Maternity collection, collated by the MoH, contains antenatal and postnatal event data obtained from primary maternity services and the NMDS. National counts of the number of women giving birth by maternal age and prioritized ethnicity, as recorded in the National Maternity collection, are available from the MoH website [25] (www.health.govt.nz; accessed 13 February 2019). Annual summaries of the estimated resident population (ERP) and number of live births, categorized by age group and total response ethnicity, were obtained from the Statistics NZ website (www.stats.govt.nz; accessed 13 February 2019).

### Statistical analysis

EpiSurv and NMDS data were imported into the statistical software ‘R’ *(R version 3.5.3 (2019-03-11))* for cleaning and analysis. A dataset of pregnancy-associated cases, with each case comprising information on the pregnant woman/mother and foetus/neonate, was created by matching EpiSurv and NMDS data primarily on the time (date of hospital admission, notification or disease onset) and geographical location (District Health Board) of the event as this information was most consistently recorded. A dataset of cases in children aged 29 days to < 15 years was created by matching EpiSurv and NMDS data by date of birth. Matched cases were confirmed by comparing patient NHI, or when NHI was not recorded or available, patient date of birth or date of delivery. Patient characteristics were derived by combining date of birth, gender, and ethnicity information from both datasets. For cases with multiple ethnicities, ethnicity was prioritized according to the order: Māori, Pacific, Asian, then European/Other. For example, a case was labelled as Māori if either EpiSurv or the NMDS recorded them as such. Patient outcomes were considered to have occurred if recorded in either EpiSurv or the NMDS.

Crude incidence rates were calculated by dividing the total number of pregnancy-associated cases or childhood cases across the 20 year period by the sum of the annual number of women giving birth or estimate resident population of children aged < 15 years respectively. Crude rates by age group and ethnicity were calculated with 95% binomial ‘Wilson’ confidence intervals (CI). Generalized linear models followed by a likelihood ratio tests (LRT) were used to assess differences across categories and calculate crude and age adjusted incidence rate ratios (IRR) with 95% CI.

### Ethics approval

This study was approved by the Central Health and Disability Ethics Committee, ethics reference number 17/CEN/227. Data were de-identified once pregnancy status was confirmed/disproved (as above) and once cross-referencing was completed. Informed consent was not required due to the retrospective nature of data collection.

## Results

In the 20 year period from 1997 to 2016, there were 147 identifiable cases of pregnancy-associated listeriosis in NZ, of which 106 (72%) were notified to ESR (EpiSurv). Hospital admissions for listeriosis were recorded in the NMDS for 88 pregnant women (60%, *n* = 47 of whom were notified to ESR) and for fourteen neonates (all of whom were notified to ESR). In addition, there were 22 cases in children aged 28 days to less than 15 years, of whom, 20 (91%) were notified to ESR and 19 (86%) were recorded in the NMDS.

### Pregnancy-associated listeriosis

Pregnant women in the sample had a mean age at time of disease onset of 30 years (standard deviation [sd] =6.5 years). Of the 144 women with prioritised ethnicity recorded, 13.2% were of Māori ethnicity, 28.5% were Pacific, 17.4% were Asian, and 41.0% were of European or other ethnicity. The gestation at onset was recorded for 59.9% of women, with a mean of 28.7 weeks (sd =7.9 weeks).

**Fig. 1:**
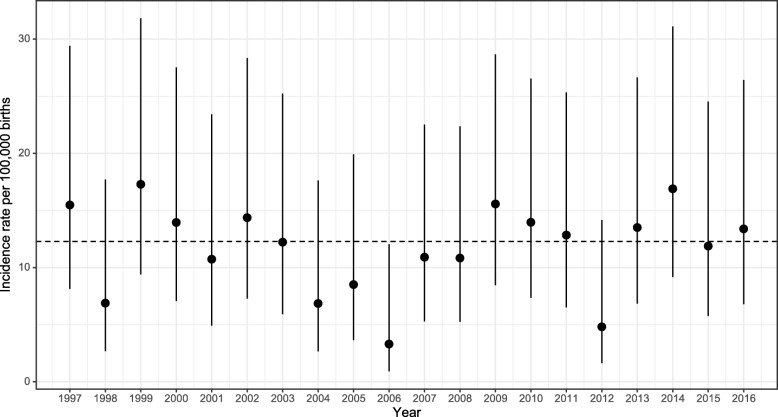
Incidence rate of listeriosis per 100,000 births in pregnant women from 1997 to 2016. Dots represent crude incidence rates per 100,000 births, and vertical lines 95% binomial confidence intervals. The dashed horizontal line represents the crude incidence rate for the entire period (1997 to 2016)

The overall incidence of pregnancy-associated listeriosis over the study period was 12.3 (95% CI 10.4 to 14.4) per 100,000 births. Incidence rates were highest in 2014 and lowest in 2006, but there was no evidence of trends over time (*p* = 0.90, Fig. 1). The estimated incidence generally increased with age, however confidence intervals were wide and there was no evidence of an association (likelihood ratio test [LRT], *p* = 0.16; Table 1). Incidence rates did differ by prioritized ethnicity (LRT, *p* <  0.001). Using denominator data as recorded in the MoH maternity collection, women of Pacific ethnicity had a rate 3.6 times higher (95% CI 2.3 to 5.6, *p* <  0.001) than women of European or other ethnicity, and women of Asian ethnicity had an incidence rate 2.3 times higher (95% CI 1.3 to 3.8, *p* = 0.002). There was no evidence of a difference in incidence rates between women of Māori versus European or other ethnicity (*p* = 0.49). A similar pattern of results was obtained when birth registrations were used as denominators, although rates for women of European or other ethnicity were lower and thus IRR tended to be larger (data not shown). Differences by ethnicity were not materially affected by adjustment for age (data not shown).

**Table 1:** Pregnancy-associated listeriosis 1997 to 2016, characteristics and incidence

Characteristic of mother	Total casesn (%)	Women giving birth (× 1000) ^b^	Incidence per 100,000 (95% CI)	IRR (95% CI)	*p* value
Age (*n* = 147)					
15 to 19	7 (4.8)	81	8.7 (4.2, 17.9)	1	*0.16*
20 to 24	22 (15.0)	214	10.3 (6.8, 15.6)	1.19 (0.53, 3.00)	0.69
25 to 29	39 (26.5)	312	12.5 (9.1, 17.1)	1.44 (0.69, 3.53)	0.37
30 to 34	35 (23.8)	352	9.9 (7.2, 13.8)	1.15 (0.54, 2.82)	0.74
35 to 39	26 (17.7)	196	13.3 (9.1, 19.5)	1.53 (0.70, 3.83)	0.32
40 to 45	11 (7.5)	43	25.6 (14.3, 45.8)	2.95 (1.16, 8.02)	0.025
Unknown	7				
Prioritised ethnicity (*n* = 115) ^a^					
European or other	44 (29.9)	511	8.6 (6.4, 11.6)	1	*< 0.001*
Māori	17 (11.6)	240	7.1 (4.4, 11.3)	0.82 (0.46, 1.41)	0.49
Pacific	33 (22.4)	108	30.6 (21.8, 43.0)	3.56 (2.25, 5.57)	< 0.001
Asian	21 (14.3)	106	19.7 (12.9, 30.2)	2.29 (1.34, 3.81)	0.002
Unknown	0				
Overall	147	1197	12.3 (10.4, 14.4)		

*P* values in italics represent overall likelihood ratio test for the age or ethnicity. All other *p* values represent Wald pairwise comparisons with reference group

^a^Ethnicity is prioritised in the order Maori, Pacific, Asian, European or other. Comparisons by ethnicity are restricted to years 2001 to 2016 due to availability of denominator data

^b^Births as recorded in the NZ MoH Maternity Collection

*IRR* Incidence rate ratio

All pregnant women with listeriosis were recorded as having been hospitalized, either in the NMDS (*n* = 88) and/or on EpiSurv (*n* = 104). There were two recorded deaths, both women were of Pacific ethnicity and reported as having died due to the listeriosis.

Foetal outcomes were recorded in all but three pregnancies (2.0%). Over a quarter (27.1%, *n* = 39) of cases were resolved in the antepartum period without the death or immediate delivery of the foetus, one third (34.0%, *n* = 49) resulted in intrauterine death or stillbirth, and the remainder in a live birth (38.9%, *n* = 56).

On average, stillborn infants were delivered at 24 weeks gestation (sd = 6.8 weeks), and liveborn infants at 35 weeks gestation (sd = 4.1 weeks). Two thirds (67.9%, 38/56) of the liveborn and 85.7% (42/49) of the stillborn infants were delivered prior to 38 weeks of pregnancy.

Of the 56 live born infants, one was recorded in EpiSurv as having died 2 days after birth (it cannot be determined if this death was due to listeriosis). Whilst half the mothers (*n* = 28) of the live born infants were recorded in the NMDS, only 14 neonates were themselves recorded in the NMDS with an ICD code for listeriosis. Nine of these neonates were admitted immediately upon birth, with the remaining five having been born elsewhere. Neonates had a mean admission weight of 2320 g (sd = 827 g) and 11 (79%) were reported has having had meningitis. None of the 14 neonates admitted to hospital were recorded as having died.

### Listeriosis in children

Between 1997 and 2016, there were 22 children between the ages of 29 days and < 15 years identified with listeriosis, giving an overall incidence rate of 0.12 per 100,000 (95% CI 0.08 to 0.19). Incidence rates were highest in infants aged less than 1 year (Table 2). There was some indication of higher rates in children of Pacific and Asian ethnicity but confidence intervals were wide due to small numbers. Eight children were recorded as having an underlying illness, with six having lymphoid leukemia.

**Table 2:** Listeriosis in children aged 28 days to < 15 years from 1997 to 2016, characteristics and incidence

Characteristic of child	Total cases, count (%) *n = 22*	Person-years (× 1,000,000)^b^	Incidence	IRR (95% CI)	*p* value
Age (years)					
29 days to < 1 year	6 (27)	1.19	0.51 (0.23, 1.10)	1	*0.006*
1 to 4	8 (36)	4.75	0.17 (0.09, 0.33)	0.33 (0.12, 1.01)	0.042
5 to 9	4 (18)	5.97	0.07 (0.03, 0.17)	0.13 (0.03, 0.46)	0.002
10 to 14	4 (18)	5.99	0.07 (0.03, 0.17)	0.13 (0.03, 0.46)	0.002
Sex					
Female	10 (45)	8.72	0.11 (0.06, 0.21)	0	0.759
Male	12 (55)	9.18	0.13 (0.07, 0.23)	1.14 (0.49, 2.70)	0.759
Ethnicity ^a^					
European or other	7 (32)	10.29	0.07 (0.03, 0.14)	1	*0.117*
Māori	5 (23)	4.34	0.12 (0.05, 0.27)	1.69 (0.50, 5.30)	0.369
Pacific	4 (18)	1.65	0.24 (0.09, 0.62)	3.56 (0.93, 11.78)	0.043
Asian	4 (18)	1.62	0.25 (0.10, 0.63)	3.63 (0.95, 12.01)	0.040
Unknown	2				
Overall	22	17.90	0.12 (0.08, 0.19)		

^a^Ethnicity is prioritised in the order Maori, Pacific, Asian, European or other

^b^Person years calculated as the sum of the estimated resident population (ERP) for years 1997 to 2016

*IRR* Incidence rate ratio. *P* value derived from likelihood ratio test (*italics*) or comparison with reference group

All but one child had hospitalisation status recorded (all these were hospitalised), but no children were recorded as having died. Fifteen children (68%) were recorded as having had meningitis and 10 (46%) septicaemia (five had both meningitis and septicaemia, and two had neither).

## Discussion

Listeriosis is a rare condition in NZ among pregnant women and children. There was no marked change in the incidence of listeriosis in women and children during the 20 year study period. Further, there were no clear trends in the incidence of disease; peaks and troughs were sporadic. This was the case for both the notification and hospitalization datasets. These findings are consistent with international literature that suggests *L. monocytogenes* is more commonly associated with sporadic episodes and outbreaks rather than being affected by factors such as climate and season [4–6].

While we identified relatively few cases of listeriosis in children, the clinical presentations were frequently of severe disease. In a study of 722 cases of listeriosis in Britain [17], spontaneous abortion occurred in 10–20% of cases, approximately 50% delivered pre-term and intrauterine foetal death occurred in approximately 11% of cases. This emphasizes the need to include cases occurring in pregnancy when assessing the true burden of disease in children.

*Listeria spp*., including *L. monocytogenes*, are ubiquitous bacteria within the NZ food supply. A 2017 microbiological survey of pre-packaged, ready-to-eat meats available in NZ reported that 15.5% were contaminated with *listeria* species [26]. Despite this, outbreaks of listeriosis in NZ are rare. Between 1997 and 2016 there were only three outbreaks recorded in EpiSurv. These occurred in the years 2000, 2009 and 2012 and involved a total of 15 cases (including cases of all ages and genders), with suspected food including cooked ham, smoked fish, and read-to-eat meat products. In addition, we identified a cluster of five pregnancy-associated cases in early to mid-September of 2009. ESR reported that these cases were possibly related, but no common food source was identified [27].

The NZ Ministry for Primary Industries produce a guidance document on *L. monocytogenes* management for industry. The guidance includes microbiological limits for *L. monocytogenes*, management of contamination, hazard analysis, shelf-life information and food safety documentation [28]. Hence, the low incidence in NZ might, in part, be due to the strict regulatory control of this pathogen within industry.

The incidence of clinical disease in pregnant women in NZ was low during the study time frame and is lower than rates reported internationally. However, the rates of listeriosis in pregnancy found in this study are still many times higher than those in the general population [22]. The NZ food safety in pregnancy guidelines include a range of information, including foods to avoid in pregnancy and safe food handling advice. On most points, these NZ guidelines are consistent with those in Australia, the United Kingdom and the United States; differences in guidance typically reflect cultural eating patterns.

This study identified for the first time that pregnant women of Pacific ethnicity have the highest risk of listeriosis. Their rates of disease were three times higher than in NZ European women. Pacific women in NZ have the highest fertility rate when compared to all other ethnicities in NZ (3.0 births per woman compared to 1.9 for European women) [29]. While fertility rate is a likely contributor to this apparent increased risk compared to other ethnic groups, a study on the nutrition intake of Pacific people living in NZ reported that Pacific people in NZ also have a higher intake of foods considered high risk for listeria when compared to other ethnic groups, for example fresh fish, shellfish, and pre-prepared foods [30]. Cultural factors around food intake, including eating during large social gatherings and communal food preparation might also play a role. This is likely to be further affected by the diversity of Pacific peoples in NZ as there are a number of distinct Pacific groups, each having their own customs related to food. More research will be required to determine the possible reasons for this, but consideration should be given to specifically targeting food safety messages to Pacific communities in NZ.

The impact of both the changing food environment and population profile must be taken into account when considering the potential impact of this disease in the future. With respect to the food environment, ready-to-eat foods, foods stored at refrigeration temperatures and foods with increased shelf life are the main sources of listeriosis. Whilst *L. monocytogenes* has been present within the food chain for at least the past 30 years, food production and intake has changed, and is ever-changing. There is now increased availability of chilled, and long shelf life ready-to-eat foods [28]. Further, the population profile must also be considered, and particularly the Pacific population which is a young, and growing population with high fertility rates.

### Strengths and limitations

NZ has a robust disease notification system regulated under the Health Act 1956. Notification can be initiated on the basis of *‘clinical suspicion’* by a medical practitioner but is usually based on the isolation of a pathogen from a clinical sample. All admissions to hospital are recorded and data on hospital admissions collated and held nationally. This study has used data from the two most comprehensive national datasets in its analysis.

Various factors influence disease notification and therefore incidence rates. Where illness is less severe, cases are less likely to consult a medical practitioner and, even if diagnosed, are less likely to be notified without laboratory confirmation. Issues associated with the cost of healthcare might also determine whether people visit healthcare providers for diagnosis. Underreporting and misclassification of listeriosis may limit our study’s findings. For example, miscarriage due to listeriosis might be missed, and maternal cases might not be diagnosed due to the non-specific nature of disease presentation.

Rates might be distorted because of errors in population estimates. Because estimates of the ‘at-risk’ population factor into the denominator for rate calculations, such errors can lead to biased estimates of incidence. In the present study, for example, only women aged 15 to 45 were considered ‘at risk’ for pregnancy and hence our estimations rely on the accuracy of national-level data collection. This might be further exacerbated by the small sample of cases.

The use of prioritized ethnicity was a limitation of this study and may have resulted in an undercount in the number of cases for those of Pacific or Asian ethnicity.

## Conclusion

NZ has a low incidence of listeriosis in pregnant women and children, however, pregnant women have much higher rates of infection than children and the general population. In addition, the consequences of infection during pregnancy and in neonates are often severe, with a high proportion of infants presenting with meningitis. Women and children of Pacific ethnicity have higher rates of disease and future public health messaging around food safety in pregnancy should specifically target Pacific communities in NZ. The severity of clinical disease, and the high case-fatality rate associated with *L. monocytogenes* infection emphasizes the critical importance of maintaining effective control measures against this pathogen. This study provides an important insight into the epidemiology of listeriosis in pregnant women and children in NZ.

## Data Availability

The data that support the findings of this study are available from The Institute of Environmental Science and Research Ltd. (ESR) and The Ministry of Health (NMDS) but restrictions apply to the availability of these data, which were used under license for the current study, and so they are not publicly available. Data are however available from the authors upon reasonable request and with permission of The Institute of Environmental Science and Research Ltd. (ESR) and The Ministry of Health (NMDS).

## Abbreviations

EpiSurv: The national notifiable disease database
ESR: The Centre for Environmental Science and Research Ltd.
ICD-9-CM: International Classification of Diseases – Clinical Modification 9
MoH: Ministry of Health
NHI: National Health Index Number
NMDS: National Minimum Dataset
NZ: New Zealand
